# "Should I and Can I?": A mixed methods study of clinician beliefs and attitudes in the management of lifestyle risk factors in primary health care

**DOI:** 10.1186/1472-6963-8-44

**Published:** 2008-02-26

**Authors:** Rachel A Laws, Sue E Kirby, Gawaine P Powell Davies, Anna M Williams, Upali W Jayasinghe, Cheryl L Amoroso, Mark F Harris

**Affiliations:** 1 Centre for Primary Health Care & Equity, University of New South Wales, Sydney NSW 2052, Australia

## Abstract

**Background:**

Primary health care (PHC) clinicians have an important role to play in addressing lifestyle risk factors for chronic diseases. However they intervene only rarely, despite the opportunities that arise within their routine clinical practice. Beliefs and attitudes have been shown to be associated with risk factor management practices, but little is known about this for PHC clinicians working outside general practice. The aim of this study was to explore the beliefs and attitudes of PHC clinicians about incorporating lifestyle risk factor management into their routine care and to examine whether these varied according to their self reported level of risk factor management.

**Methods:**

A cross sectional survey was undertaken with PHC clinicians (n = 59) in three community health teams. Clinicians' beliefs and attitudes were also explored through qualitative interviews with a purposeful sample of 22 clinicians from the teams. Mixed methods analysis was used to compare beliefs and attitudes for those with high and low levels of self reported risk factor management.

**Results:**

Role congruence, perceived client acceptability, beliefs about capabilities, perceived effectiveness and clinicians' own lifestyle were key themes related to risk factor management practices. Those reporting high levels of risk factor screening and intervention had different beliefs and attitudes to those PHC clinicians who reported lower levels.

**Conclusion:**

PHC clinicians' level of involvement in risk factor management reflects their beliefs and attitudes about it. This provides insights into ways of intervening to improve the integration of behavioural risk factor management into routine practice.

## Background

Smoking, poor nutrition, excessive alcohol consumption and lack of physical activity are the main behavioural risk factors for chronic disease [1], which accounts for more than 60% of the overall global burden of disease now, and an expected 80% by the year 2020 [2]. Primary health care (PHC) clinicians often have contact with patients over an extended period of time, which provides important opportunities for assessing for lifestyle risk factors, developing action plans, monitoring progress and referring to community support programs if required [3]. This includes general practitioners, nurses, allied health clinicians and other health workers such as multi-cultural and indigenous health workers, often working in multi-disciplinary teams [4]. The 5As model of brief intervention (ask, assess, advise, assist, arrange) has been shown to be an effective approach to behavioural risk factor management in primary health care [5]. There is however a gap between opportunity and practice, with relatively low rates of intervention [6,7]. This highlights the need for better understanding of how risk factor management can be integrated into routine PHC services [8].

Risk factor management has been shown to be related to clinicians' beliefs and attitudes, and also to health service structure and organisation. Clinician self-efficacy [9-13], personal lifestyle behaviours [10,14-17], perceived effectiveness of interventions [9,13-15,18-20] and perceived congruence with clinician role [9,15,17,20] have consistently been shown to be important in influencing the management of lifestyle risk factors, along with perceived patient motivation [12,13,17,18] and concern regarding client acceptance [15,17,20]. Most studies have focused on general practice, although studies of PHC nurses in the USA and Finland have highlighted the importance of clinician beliefs and attitudes in understanding smoking cessation practices. Clinician self efficacy [13,21], perceived effectiveness [13,16,18], beliefs about client receptiveness [21] and clinicians' smoking status [13,16,21] in particular were found to be associated with the likelihood of providing smoking cessation advice.

To date, studies have been largely descriptive [11,12,14,15,17-20,22] or cross sectional, and have generally investigated the management of individual behavioural risk factors [9,10,13,16,21]. They have also tended to report qualitative and quantitative findings separately rather than link quantitative data of actual practice to qualitative data on beliefs and attitudes related to such practices.

The aim of this mixed methods study was to explore the beliefs and attitudes of PHC clinicians related to the management of lifestyle risk factors and examine differences between those with high or low levels of risk factor management practices. Combining quantitative data about levels of risk factor intervention with qualitative data about beliefs and attitudes was expected to suggest ways in which interventions might be designed to increase the overall rate of risk factor management.

## Methods

This paper presents findings from a baseline assessment of three community health teams participating in a feasibility study to test interventions to incorporate lifestyle risk factor management into routine practice. The mixed methods study design was informed by a pragmatic epistemology [23] where data from a cross sectional survey on reported engagement of risk factor management activities (high or low) were linked to semi-structured interviews exploring clinician views of risk factor management practices. The combination of the two approaches provides a more complete picture than either could on its own [23].

### Description of participating teams/services

The project involved three community health teams from two Area Health Services in the state of New South Wales (NSW), Australia. In NSW, Area Health Services are responsible for providing all hospital and community based health care apart from general practice, which is funded by the Commonwealth Government. Community health services are the second largest provider of publicly funded PHC services to the general population after general practitioners (GPs) [24]. Community health teams were selected for the study through expressions of interest. Team one (n = 35) was a generalist community nursing team with both enrolled and registered generalist community nurses, located in a metropolitan area (see appendix 1 for a description of the educational qualifications and role of registered versus enrolled nurses involved in the project). Team two (n = 16) was a co-located multi-disciplinary community health team from a rural area. This team consisted of community nurses, child and family nurses and allied health staff. Team three (n = 10) consisted of PHC nurses, Aboriginal health workers, and allied health clinicians providing PHC services to rural and remote communities that generally did not have access to other health services such as a GP.

### Clinician survey

All clinicians in the participating teams were invited to complete a short 20 item self-administered survey at a team meeting to assess current risk factor management practices, perceived knowledge, confidence and attitudes. They were asked to report the proportion of new and review clients seen over the past 2 weeks who they: 1) asked about smoking, nutrition, alcohol and physical activity; 2) assessed for readiness to change; 3) provided verbal and written advice on these risk factors; 4) referred to other services for support in changing risk factors; 5) followed up progress in subsequent visit(s). The survey also asked about knowledge and confidence in screening and managing each risk factor. Attitudinal measures for each risk factor included: perceived effectiveness of intervention; perceived importance to health; perceived importance for clients seen; perceived work priority and perceived client acceptability of raising the lifestyle risk factor. All items were measured on a 5 point Likert scale. The survey [25] was adapted from a previous survey developed to assess GP risk factor management practices and capacity [26]. It was reviewed by the project team and community health managers for face validity and piloted with a community health team not involved in the study.

The results were analysed using SPSS statistical software (version 14; SPSS, Chicago, IL, USA). The relationship between clinician practices (dependent variables) and perceived confidence, knowledge and attitudes (independent variables) was analysed initially using univariate logistic regression. Because of the relatively small sample of clinicians, dependent variables were recoded as low, moderate or high to increase the number of responses in each category. Independent variables found to be significantly related to practices in univariate analysis (at the 0.01 significance level) were then entered into multivariate logistic regression models. Due to the large number of tests undertaken, results are treated as significant at the (P < 0.01) level for univariate the analyses, and (P < 0.05) in the multivariate analyses.

### Semi- structured interviews

Following the survey face to face interviews were conducted with a purposeful sample of clinicians from the three teams to provide an in-depth understanding of the barriers and enablers they perceived to addressing lifestyle risk factors as part of their routine work. Clinicians were either invited to express interest in participating in an interview at a team meeting (team one) or were approached individually by the local project officer to take part (teams two and three). The aim was to recruit a sample of clinicians who varied in profession and role (enrolled and registered nurses, allied health staff and Aboriginal Health Workers), experience and geographical location.

Interviews were conducted by the Project Leader and covered issues related to barriers, enablers and capacity to address lifestyle risk factors as part of routine practice (Table 1). Interviews were audio-taped with participants' permission and transcribed verbatim for thematic analysis. The Project Leader identified and coded themes using NVivo 7.0 software, based on repeated reading of the transcripts and coding of issues of interest to the research question [27]. Two authors then read the transcripts to confirm the themes, and key findings were reviewed in feedback sessions with each team. This paper reports themes related to clinician attitudes and beliefs, and relates them to self reported levels of risk factor management.

**Table 1:** Interview topic guide for clinicians

• Overview of job role
• How addressing SNAP risk factors fits with job role
• Approach to addressing SNAP risk factors in job role
• Work priority to address SNAP risk factors
• Confidence to address SNAP risk factors
• Barriers and enablers to addressing SNAP risk factors in routine work
• Support and resources required to address SNAP risk factors in routine work
• Role in supporting generalist staff to address risk factors (allied health staff only)
• Ability to accept referrals from generalist staff (allied health staff only)

SNAP: smoking, nutrition, alcohol and physical activity

### Mixed methods analysis

Data for those clinicians who participated in an interview was linked to their survey data to gain a deeper understanding of their risk factor management practices. Results from the interviews (n = 22) were compared between clinicians reporting high and low levels of risk factor management (high and low implementers). High implementers were defined as clinicians with total screening and/or intervention scores (across all risk factors) in the fourth quartile for clinicians participating in an interview. Low implementers were those with total screening and/or intervention scores less than or equal to the first quartile out of those participating in interviews. Screening scores were calculated as the sum of the Likert scale for the proportion of new clients asked about each risk factor of the previous 2 weeks and intervention scores were calculated as the sum of the Likert scale for intervention activities for each risk factor. Implementation status (high or low) was recorded in NVivo 7.0 software as an attribute, allowing coded themes to be extracted separately and compared for high and low implementers.

### Ethics

The study was approved by the UNSW Human Research Ethics Committee (HREC) and the HREC in each Area Health Service.

## Results

A total of 59 out of the 61 clinicians completed the survey (96.7% response rate) and interviews were conducted with 22 of these clinicians (team 1: n = 7, team 2: n = 11, team 3: n = 4). Out of those interviewed, five clinicians were classified as high implementers and five were classified as low implementers. Clinicians participating in the interviews were broadly representative of those completing the survey (Table 2). The majority of clinicians were female, aged 45 to 55 years, with a wide range of professional experience. Key themes relating to clinician attitudes and beliefs towards the management of lifestyle risk factors are described below. For each theme, qualitative data was compared for low and high implementers (Table 3). Qualitative findings were also triangulated with results from a cross sectional analysis of the survey data (Table 4).

**Table 2:** Characteristics of the clinicians participating in the survey and interviews

	**Survey(n = 59)**	**Interviews (n = 22)**
**Age Category, No. (%)**		
18–24 years	2 (4)	2 (9)
25–34 years	6 (11)	2 (9)
35–44 years	16 (29)	6 (27)
45–54 years	25 (45)	11 (50)
55–64 years	7 (13)	1 (5)
		
**Clinician experience, mean (std), range**		
Years in profession	22 (11), 1 – 46	21 (11), 1–35
Years in community health	10 (8), 0.4 -30.0	8 (6), 0.4–16
Years in team	6 (6), 0.03 -22.0	7 (6), 0.4–16
		
**Gender, No (%)**		
Male	4 (7)	0 (0)
Female	55 (93)	22 (100)
		
**Employment, No (%)**		
Part time	26 (48)	10 (45)
Full time	28 (52)	12 (55)
		
**Clinician type, No (%)**		
Registered nurse	40 (68)	16 (73)
Enrolled nurse	10 (17)	1 (5)
Allied health	9 (15)	5 (23)

Unknowns for survey data were: age-3 ; employment-5

**Table 3:** Comparison of key belief and attitude themes for high and low implementers

**Theme**	**High Implementers**	**Low Implementers**
**Congruence with clinician role**	▪Risk factors perceived to be relevant to the presenting issues and types of clients seen. ▪Opportunity to address as part of routine care.	▪Risk factors perceived to be less relevant to the clinicians' role and reason for seeing the client. ▪Confusion over role boundaries, uncertainty about whether risk factors should be addressed by another health professional.
**Perception of client acceptability**	▪Opportunity to develop rapport with clients. ▪Linked discussion of risk factors to the presenting issue. ▪Undertook screening for risk factors as part of standard assessment. ▪Flexible approach to intervention.	▪Less able to link discussion of risk factors to presenting issue. ▪Concerned about being seen as judgemental, receiving a negative reaction from clients and damaging clinician-client relationship.
**Beliefs about capabilities**	▪High perceived confidence and comfort in addressing risk factors.	▪Expressed a lack of knowledge, skills or confidence in addressing risk factor issues in an appropriate way.
**Perceived Effectiveness of risk factor intervention**	▪Low perceived effectiveness but responsibility to provide intervention as part of the role. ▪Value of intervention recognised but difficulty in assessing and measuring outcomes.	▪Low perceived effectiveness of risk factor intervention.
**Clinicians' own lifestyle**	▪Own lifestyle habits acted as an enabler for some (eg ex-smoker) or did not influencing risk factor management practices	▪Own lifestyle habits identified as a barrier for some in addressing risk factor issues with clients.

**Table 4:** Factors associated with risk factor management practices- survey data

**Variable**	**Univariate Analysis Odds Ratio (CI)**	**Multivariate Analysis Odds Ratio (CI)**
**SMOKING**		
**Ask new clients about smoking**		
Perceived client acceptability- low	1.00 (reference)	1.00 (reference)
Perceived client acceptability- high	8.9* (1.7–47.3)	13.1* (1.0–174.6)
**Follow up progress**		
Knowledge of adult education principles -low	1.00 (reference)	1.00 (reference)
Knowledge of adult education principles -moderate	18.7** (1.9–184)	NS
Knowledge of adult education principles -high	19.3** (2.1–178)	NS
Confidence in assessing nicotine dependency- low	1.00 (reference)	1.00 (reference)
Confidence in assessing nicotine dependency- high	16.5** (2.7–101.3)	NS
Confidence in discussing smoking recommendations-low	1.00 (reference)	1.00 (reference)
Confidence in discussing smoking recommendations-high	7.6** (1.7–34.9)	NS
		
**NUTRITION**		
**Ask new clients about nutrition**		
Clinician type – allied health	1.00 (reference)	1.0 (reference)
Clinician type – registered nurse	12.0* (1.3–106)	571.7* (2.4–135,890)
Confidence in assessing nutrition – low	1.00 (reference)	1.00 (reference)
Confidence in assessing nutrition – high	4.9** (1.5–15.5)	NS
Confidence in discussing nutrition recommendations – low	1.00 (reference)	1.00 (reference)
Confidence in discussing nutrition recommendations – high	6.8** (2.1–22.4)	NS
Work priority – low	1.00 (reference)	1.00 (reference)
Work priority – moderate	6.5** (1.1–38.6)	NS
Work priority – high	7.8** (1.8–34.1)	NS
**Time spent discussing nutrition**		
Knowledge of nutrition recommendations – low	1.00 (reference)	1.00 (reference)
Knowledge of nutrition recommendations – high	5.8** (1.0–17.9)	NS
		
**ALCOHOL**		
**Ask new clients about alcohol**	1.00 (reference)	1.00 (reference)
Perceived client acceptability – low	9.8** (2.2–44.1)	8.0* (1.3–46.2)
Perceived client acceptability – high		
		
**PHYSICAL ACTIVITY (PA)**		
**Ask new clients about PA**		
Perceived client acceptability – low	1.00 (reference)	1.00 (reference)
Perceived client acceptability – high	11.5** (2.8–47.6)	9.7* (1.4–65.7)
Confidence in assessing PA – low	1.00 (reference)	1.00 (reference)
Confidence in assessing PA – high	4.7** (1.5–14.9)	NS
Confidence in discussing PA recommendations- low	1.00 (reference)	1.00 (reference)
Confidence in discussing PA recommendations- high	7.6** (2.2–26.4)	NS
**Verbal advice about PA**		
Perceived client acceptability – low	1.00 (reference)	1.00 (reference)
Perceived client acceptability – high	21.9** (2.3–187.0)	17.6* (1.6–189.5)
**Time discussing PA**		
Confidence in discussing PA recommendations- low	1.00 (reference)	1.00 (reference)
Confidence in discussing PA recommendations- high	5.2** (1.6–16.4)	NS
Work priority – low	1.00 (reference)	1.00 (reference)
Work priority – high	9.2** (2.0–41.7)	NS
**Follow up progress with PA**		
Knowledge of PA assessment- low	1.00 (reference)	1.00 (reference)
Knowledge of PA assessment -high	12.5** (2.4–64.0)	NS

* P < 0.05, **P < 0.01, NS: non significant. Only univariate results were P < 0.01 are reported in the table.

### Clinician characteristics

Four out of five high implementers were registered nurses with professional experience ranging from 23 to 33 years. In contrast the low implementer group consisted of two registered nurses, one enrolled nurse and two allied health professionals (experience ranging from one to 33 years). In line with these findings the survey analysis showed that registered nurses were more likely to ask about nutrition compared to allied health professionals, however clinician type or years of experience was not associated with any other risk factor management practices (Table 4).

### Congruence with clinician role

Clinicians' perception of how well risk factor management fitted with their role was an important theme, with differences between high and low implementers. High implementers reported risk factors as being directly relevant to their clients and felt there was adequate opportunity to address these as part of routine care. "*a large part of our work is wound care, so in some ways I think this sort of thing fits right in because of the whole smoking, nutrition, mobility, exercise, all impact greatly on wound care" *(High implementer, team 1). By contrast low implementers did not see that they had a role in addressing risk factors with clients, or felt their was some confusion over role boundaries and whether risk factors might be addressed by another health professional: *"I don't know...whether it should be something we address, or whether we feel it's addressed to by other people...we know that they're dealing with their doctor, and often, a community nurse is involved, and a lot of the clientele we see are serviced by other professionals" *(Low implementer, team 2).

This is consistent with the results of the univariate analysis of the clinician survey, which also showed a relationship between clinician ratings of work priority and some risk factor practices. Clinicians who rated nutrition as a moderate or high work priority were significantly more likely to report asking clients about nutrition, and those rating physical activity as a high work priority also reported spending longer discussing physical activity with clients (Table 4). These findings did not remain significant in multivariate analysis. No association was found for smoking and alcohol.

### Perception of client acceptability

Client acceptance of risk factor management was also an important theme. All clinicians saw client acceptance of lifestyle interventions as important and identified developing rapport with the client as critical for being able to discuss lifestyle issues. However there were some interesting differences in how high and low implementers viewed these issues. High implementers generally considered that they had the opportunity to develop rapport with clients and some reflected on positive reactions that they had from clients in raising lifestyle issues. *"Often they're aghast, somebody is actually taking an interest in my health, let me tell you all about it!" *(High implementer, team 3). They appeared to use a number of strategies to improve client acceptance, including linking the discussion of risk factors to the presenting issue, explaining the rationale for risk factor screening and making this part of the standard assessment process, being sensitive to clients' reactions and tailoring their approach to each individual. "*You explain it to them while you're having a chat with them and.. their defences drop, and they think 'oh, they're not here to bombard me, they're just interested" *(High implementer, team 1).

While low implementers also used some of these strategies, they expressed a number of concerns about client acceptance including being seen as judgmental, receiving negative reactions from clients and damaging the clinician-client relationship. "*You know, we're on their turf, that's the way I look at it. We're a guest, we're a professional guest in their home, and we can't judge social issues you know" *(Low implementer, team 1). "*If I push how many cigarettes do you have a day, you know, they'd be saying 'why are you asking me this? I'm not coming here for drug and alcohol counselling, I'm coming here for a different issue" *(Low implementer, team 2).

In the survey, perceived client acceptance was the only factor independently associated with clinicians' asking clients about smoking, alcohol and physical activity (but not nutrition) (Table 4). Perceived client acceptance was also the only factor independently predicting whether clinicians would provide verbal advice about physical activity.

### Beliefs about capabilities

Clinicians' confidence in addressing lifestyle risk factors was related to their risk factor management in both the quantitative and qualitative analyses. In the interviews, high implementers generally expressed more confidence in addressing lifestyle risk factors than did low implementers. "*I guess I'm fairly comfortable in the way that I do it. I'm not often shown the door" *(High implementer, team 1). *"I feel I've got the skills and knowledge to do it" *(High implementer, team 2). In contrast, low implementers tended to reveal a lack of knowledge/skills or confidence. "*Oh, I don't have the confidence...not through knowledge or understanding...just through the confidence to speak to the person about it *(Low implementer, team 1). Confidence was not always related to the specific risk factor, but rather to the general skills required to raise and address such issues in an appropriate way.

This was confirmed by the univariate analysis of the survey data, which showed that clinicians with greater confidence in addressing poor nutrition and physical inactivity were more likely to screen clients for these risk factors (Table 4). Confidence in discussing nutrition and physical activity recommendations was also associated with spending more time discussing these risk factors with clients. Similarly, clinicians with greater confidence in screening for nicotine dependency and discussing smoking recommendations were more likely to follow clients up after a smoking cessation intervention. These findings did not remain significant in multivariate analysis (Table 4). Perceived knowledge or confidence in screening for alcohol intake or alcohol recommendations was not associated with screening or intervention for alcohol.

### Perceived effectiveness of risk factor intervention

The effectiveness of risk factor interventions was an issue for all clinicians. They thought that interventions were less likely to be effective for older clients or those with ingrained patterns of behaviour, those with lower levels of education and those with poorer compliance in other areas of care. One clinician felt that the success of media campaigns meant that that the general population already know what they should be doing.*"People already know. If they're choosing to still embrace those things, I dare say short of seeing the six foot pine box looming at the front door. I don't know what it takes to change some peoples' minds" *(Low implementer, team 1).

There was a distinct difference in attitude between high and low implementers. Although high implementers expressed uncertainty about the effectiveness of their interventions, this did not appear to deter them from intervening as they felt it was their responsibility to do so. *"Well I'm quite sure that much advice will not be effective very, very frequently, but it doesn't mean that I can't, I mean you have a responsibility where you can to try and steer people in the right direction*" (High implementer, team 1). Other high implementers were more optimistic about the effectiveness of their interventions but thought it was difficult to assess outcomes. *"I just think it's fabulous and it's as if you make a difference, but it hard to tell" *(High implementer, team 3). Similarly, the survey analysis found no association between perceptions of effectiveness and levels of self reported risk factor management activity (Table 4). This highlights the importance of the different ways high and low implementers responded to the perception of low effectiveness and the impact that this may have on risk factor management practices.

### Clinicians' own lifestyles

Some low implementers identified their own lifestyle habits as a barrier for addressing risk factors. *" I'm not a smoker, I'm not a drinker, I'm a physically active person, so I find that I'm not the right person in myself to be addressing it*" (Low implementer, team 1). However for high implementers this was either not an issue, or was even an enabler. Two high implementers reported having changed some aspects of their own lifestyle and found this helpful when giving advice to clients. *"being an ex-smoker I feel more qualified to give them advice" *(High implementer, team 3). Other high implementers recognised that they had a lifestyle risk factor, but this did not deter them from providing intervention to others.*"because I feel I'm a little overweight, I sometimes feel a bit funny telling people what to eat...but it doesn't stop me doing it" *(High implementer, team 3).

## Discussion

This study provides new information about the beliefs and attitudes of Australian PHC clinicians outside general practice towards the management of lifestyle risk factors. The key beliefs and attitudes identified as important are in line with those reported within general practice across different lifestyle risk factors [9-12,14,15,17,19,20,22] and previous studies of the beliefs and attitudes of PHC nurses towards smoking cessation intervention [13,16,18,21]. The study also shows an interaction between clinicians' beliefs and attitudes towards lifestyle risk factor management and their practice. Role congruence and perceived client acceptability were related to whether clinicians believed they 'should' address risk factors, and their beliefs about their capabilities and the effectiveness of risk factor management were related to whether they thought they 'could' provide intervention for lifestyle risk factors. Clinicians' own lifestyle was also found to act as a barrier or enabler for some clinicians.

In a cross sectional study of this kind it is not possible to identify causal relationships between clinician beliefs, attitudes and practice. Beliefs and attitudes may influence practice, beliefs and attitudes may be expressed that are consistent with pre-existing practices, or they may mutually reinforce each other. Thus high implementers who reported using specific strategies to improve client acceptability may have been able to do this because of their strong beliefs in their capabilities (self efficacy), which may have been enhanced by their previous experience in risk factor interventions. This is supported by the quantitative survey analysis in which individual attitudes were significantly related to practice in univariate analysis but did not remain significant in multivariate analysis suggesting a high level of interaction between these variables. This was with the exception of perceived client acceptability which was the only factor independently associated with risk factor practices.

Our results extend the findings of previous studies by showing how attitudes vary between clinicians who report high and low levels of risk factor management. This provides new insights into possible strategies for encouraging risk factor management in PHC. The value placed on role coherence suggests making risk factor management interventions as consistent as possible with other clinical activities, presenting issues and ways that PHC clinicians normally work. The importance of the broader health care context and the priorities of the health care organisation [28] suggest that clinicians may be more likely to see risk factor management as part of their role if it is integrated into service policies and procedures, job descriptions, orientation for new staff and standard assessment processes. Confidence may be built through practical skill based training in behaviour change. Conducting lifestyle interventions is a relatively complex task that requires skills in counselling and behaviour change strategies often not taught in undergraduate courses [29]. It would be important to incorporate behaviour change principles in undergraduate training and provide further opportunities to develop counselling skills as part of ongoing professional development. This education should include activities designed to influence attitudes and beliefs such as group discussion, peer support and reflection on practice [30].

The key themes reported in this study are in line with a number of theoretical domains identified as important in promoting clinician behaviour change including beliefs about capabilities, beliefs about consequences (client acceptability, perceived effectiveness) and social/professional role and identity (role congruence and own lifestyle) [31]. These also reflect some of the key constructs of the theory of planned behaviour (TPB) [32]. The TPB has been used to explain intention to promote physical activity and offer smoking cessation intervention in two previous studies, explaining 61% and 40% of the variance respectively [9,33]. Seeing risk factor management as a component of the job role and believing that clients accepted risk factor interventions fits with the 'construct of subjective norm' a component of the TPB. Clinicians' beliefs about their ability to intervene and about the results of doing so also fits with the other key constructs of the TPB: 'attitudes towards the behaviour' and 'perceived behavioural control'. Previous studies [9,33] that have used the TPB to explain risk factor management practice did not include perceived client acceptability as part of the 'subjective norm' construct and our data suggest that this may help improve the application of this theory to risk factor management practices.

The study has a number of limitations. Our findings are based on a relatively small sample of PHC clinicians working in small number of community health teams. It is not certain how far these findings apply to PHC workers in other settings. The sample size was relatively small: this limited statistical power and resulted in wide confidence intervals for the quantitative findings. The survey and interview findings are based on self-report, which may not reflect actual practice and could lead to over-reporting of activities perceived as socially desirable, such as providing intervention for risk factors. Those who agreed to participate in an interview may have been more interested in risk factor management issues. Clinicians were however purposefully sampled to ensure a variation in the types of health professionals, experience, geographical location and team, providing insights from multiple perspectives. The mixed methods nature of the study also provided a means to compare qualitative and quantitative findings and provide specific insights into factors associated with risk factor practices from the perspective of high and low implementers.

There are a number of unresolved issues worthy of future research. Our data is part of an initial baseline assessment before a capacity building intervention. It is uncertain how far the barriers raised by those low implementers are hypothetical and reflect a lack of experience with addressing risk factors. It is also uncertain to what extent beliefs and attitudes may be changed by an intervention and whether these changes would result in changes in practice. The paper also does not address organisational and structural factors that have also been shown to influence health promotion and risk factor management practices [14,34,35]. It is uncertain how these factors interact with beliefs and attitudes to influence risk factor practices. Further research is also required to explore the views of clients about the acceptability of PHC clinicians screening and offering intervention for lifestyle risk factors. While research conducted in the general practice setting suggests high levels of client acceptance [36], there is still relatively little research outside of general practice.

## Conclusion

This study confirms that the beliefs and attitudes of primary health care (PHC) clinicians are important in understanding risk factor management practices. Strategies to improve such practices should consider how well risk factor management activities fit with the clinician role and address clinician confidence, beliefs about client acceptability, beliefs about effectiveness and clinicians' attitudes about the impact of their own lifestyle on practice. Further research is required to examine whether beliefs and attitudes are amenable to change overtime in response to such interventions and whether this results in changes in practice.

## Competing interests

The author(s) declare that they have no competing interests.

## Authors' contributions

RAL contributed to study design, data collection, qualitative and mixed methods analysis and wrote the first draft of the manuscript. SEK undertook the quantitative data analysis, AMW and GPD contributed to study design, qualitative data analysis, data interpretation and contribution to the manuscript, UWJ contributed to quantitative data analysis and interpretation. CA contributed to data interpretation and MFH contributed to study design and data interpretation. All authors read and approved the final manuscript.

## Appendix 1

The educational qualifications and role of registered versus enrolled nurses involved in the project.

In Australia registered nurses undertake a three year tertiary education program. Enrolled nurses undertake training from 12 months to two years at a technical college receiving a certificate or diploma depending on the state. Enrolled nurses work with registered nurses to provide patients with basic nursing care. Within the project registered nurses undertook the initial client assessment and care planning and enrolled nurses assisted with the implementation of the care plan.

## Pre-publication history

The pre-publication history for this paper can be accessed here:


